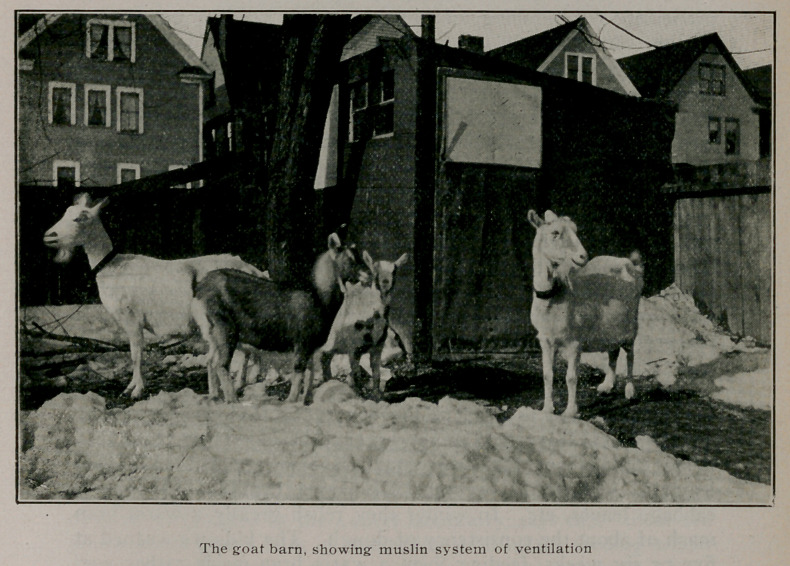# Our Back Yard Dairy

**Published:** 1908-05

**Authors:** W. Sheldon Bull


### Our Back Yard Dairy

BY W. SHELDON BULL

(From the *Good Housekeeping* Magazine. for April, 1, 1908. By permission.)

IN spite of the increase of knowledge concerning the production and care of milk, the fact remains that ordinary milk, while of almost universal use, is the most uncleanly article of food on the table." So declares Dr. C. W. M. Brown at the annual meeting of the American Medical Association in 1906. The *Lancet* of September 7, 1907, said editorially that "the second report of the royal commission on human and animal tuberculosis is of itself sufficient to put beyond any doubt the serious danger of contracting tuberculosis to which the community is daily exposed by means of the milk of tuberculous cows."

In the absence of practical protective measures on the part of the general and state governments, sufficiently comprehensive to guard the interests of the entire population, a great debt of gratitude is due the public-spirited and progressive health officers of a few of our cities, who, by rigid inspection, and by insistence upon the necessary sanitary precautions on the part of both dairymen and dealers, are doing their best, under totally inadequate laws and against tremendous odds, to insure a pure milk supply for their respective cities. The degree of protection afforded these fortunate cities is, however, at the best but small.

When the health department of one of these cities locates a tuberculous cow at a farm, in the immediate vicinity, or one fifty or one hundred miles away, the milk from that cow can be excluded only from the city concerned; it may be sent to another city or town having either an inefficient health department or none at all, or situated across the state line, and thus emperil the lives of those who consume it. The effort to protect the health of their own townspeople on the part of these comparatively few health officers is naturally not widespread enough to more than skim the filth from the top of Uncle Sam's great big milk pail.

Notwithstanding the fact that we are residents of a city so fortunate as to possess a health commissioner whose intelligent zeal and advanced and original methods in dealing with the important problem of a pure milk supply have given him a national reputation, an earnest desire to become independent of the milkman and his milkman's milk," induced us to take up the study of capriculture. The fact that the milch goat, unappreciated, neglected and much ridiculed in this country, is practically immune from tuberculosis, together with her numerous other good qualities, led us to take up the question of her availability as a source of supply of pure "homemade" milk for family use.

Dr. J. Finley Bell, in a paper, on "Some fat problems and goat's milk in infant feeding," read before the New York Academy of Medicine, claims the following, among other advantages, for the milch goat: "She is more docile, less excitable, not subject to tuberculosis or other disease in this climate. Being a browser rather than a grazer, she will thrive where cows would not; and, above all, she is cleanly. Her excrement is solid and her tail short, consequently she is not covered with manure as is the cow. It is safe to assert that the production of cow's milk free from manure bacteria is commercially impossible. Not so with the goat; she can be easily washed (tubbed, if necessary), and aproned for milking."

With a view to experimenting as to Nanny's capability for furnishing a city household with its daily milk supply, we have established a small goat dairy in our own back-yard, in the heart of the residence district of a large city.

Not a few of our friends assured us that we should find it impossible to use the milk, owing to a "goatish" taste. As none of these kind counselors had ever tasted goat's milk, we preferred

Young Saanen Doe from Switzerland to rely for authority on that subject on the statements made by those who have made a scientific study of the milch goat for many years. We quote from an English authority as follows: "Contrary to the usually accepted theory, there is no unpleasant flavor to the milk if conscientious cleanliness in the care of the goats is maintained. In countries where sterilising the milk pail and bottles and washing the hands before milking are not much thought of the milk may possibly acquire a peculiar taste." A German authority says: "An aftertaste of goat's milk, according to statement of veterinarians, should not exist, and if any such taste or smell does exist, it must be traced to unclean stables or bad feed. Even cow's milk very frequently smells badly under these conditions." Since becoming milch goat keepers we have found from actual experience that the milk is not only delicious in flavor, but that it is much richer than cow's milk.

At the beginning of our experiment we decided that, instead of investing in imported does, it would be more practical and less expensive to purchase a goat of our native common stock, good specimens of which we had been informed might be found in the outskirts or foreign quarters of our city, and very often bought for a small sum. Trips to these outlying districts, in quest of stock for the nucleus of our goat dairy, we found both novel and instructive. Having discovered a promising-looking doe, the next step was to get information in regard to her owner, her milk yield and her price. The answer to the first of these questions we usually obtained from the children of the neighborhood, who were more numerous than the goats, and who, upon the judicious bestowal of a few pennies, readily pointed out the goat owner's abode. As there is no established price for the native goat, several excursions of this kind were necessary before we found a goat just suited to our mind, and when discovered, it took some bargaining before we became the proud possessors of our first Nanny.

As she was nearly dry, we decided to postpone the date for actually establishing a home dairy, and to board the doe for the winter in the neighborhood where she was purchased. As goats are proverbially prolific, we set about securing an increase in the size of our "herd" of one by breeding Nanny to a pure-bred Toggenburg buck of imported stock.

In regard to breeding milch goats, the late Mr. George Fayette Thompson said, in a bulletin entitled Information Concerning the Milch Goat, compiled by him for the United States department of agriculture: "With goats, as with other domestic animals, it is very essential that the best buck possible be employed. One should always avoid what are usually referred to as 'common' bucks." We experienced no great difficulty in securing the services of a pure-bred buck, as a number of fine specimens of the Saanen and Toggenburg breeds were brought to this country in 1904 by several goat fanciers, who united in making the importation. There were also imported a number of Saanens, Toggenburgs and Schwartzenburg-Guggisbergers in 1905 and 1906. These goats and their off-spring have become scattered throughout the United States. Information concerning the whereabouts of these imported animals, also copies of the Thompson bulletin (No. 68), may be obtained by applying to the bureau of animal industry, United States department of agriculture, Washington.

Our breeding experiment has resulted in two fine half-bred Toggenburg kids, a buck and a doe, both hornless and unusually large, notwithstanding the fact that their mother has a well-developed pair of horns and is rather small. These kids, together with two imported pure-bred Saanen does, since purchased, now comprise the herd of our "back-yard dairy."

The Saanen milch goats are pure white or cream white in color, usually hornless, much larger and finer than our native goats, and are noted as milk producers, having been bred for centuries in Switzerland with that object in view.

In regard to the care of our goats, a suitable stable for their housing was naturally our first consideration. This was built at

The goat barn, showing muslin system of ventilation a trifling expense, being nothing more than a shed of rough, second-hand lumber, covered outside and lined throughout inside with tar roofing paper, and lighted by several windows on the south side. We use the "muslin system" of ventilation, having filled in the upper panels of the door, which is situated at the east end of the shed, and also one of the window frames on the south side, with unbleached cotton in place of glass.

We were so fortunate as to be favored before the shed was entirely completed, with a visit from Dr. E. M. Santee, assistant dairyman of the United States department of agriculture, an expert on the subject of "muslin ventilation," and in accordance with his suggestion we made the ventilating spaces larger than we had at first contemplated.

This shed of homemade construction, while not a thing of beauty, has proven to be dry, warm, light and well-ventilated. It is situated in the northwest corner of our thirty-foot front city lot, the rear forty feet of which is fenced off with wire fencing, giving the goats an inclosure for exercise in pleasant weather, and yet keeping them from nibbling the plants and shrubbery in the forbidden territory of lawn and flower bed next to the house.

A long plank, resting on two posts set in the ground, and an old crate with a plank leaning up against it, satisfies the kids' propensity for climbing and jumping. An outdoor gymnasium of this sort not only affords an opportunity for much needed exercise on the part of the growing kids, but also furnishes much amusement to anyone who has the time to watch the antics of these graceful and playful little animals. A quantity of brushwood heaped about the shade trees situated in the rear of the goat yard affords an opportunity for browsing and bark-peeling, while protecting the bark of the tree trunks from the caprine teeth.

While constitutionally a very hardy animal, the domesticated goat is more delicate than in its wild state, and while of all domestic animals the least liable to disease, still it is very susceptible to sharp winds, cold rains or mud. Although our environment makes it impossible for us to give our goats the run of a pasture, we have no trouble in keeping them in perfect health while stall fed by having the yard for their exercise.

We feed them three or four times a day, according to the time of year, only as much as they eat at one feeding being given. This we soon learned to gauge by experience. Their chief feed is hay, supplemented by bran, grain, vegetables and clean kitchen leavings, such as the peelings of potatoes, turnips and parsnips, cabbage leaves, etc. In winter they relish greatly a warm bran mash of about the consistency of dough. The kids we weaned at five or six weeks, feeding them a warm bran mash, rather wet, and later on some green stuff, grain and hay.

With reference to the feed, the methods of feeding, the interior arrangement of the shed, and the care of the goats in general, we have followed, so far as possible, the practice advised by Crepin, the French, Pegler and Hook, the English, and Dettweiler, the German, authorities deriving much pleasure and profit from our study of the works of these authors.

The hay is fed from small hayracks over each little manger, the bars or slats of the hayrack being close enough together to prevent the goats from pulling down more than a mouthful at a time. They are such fastidious creatures that they will refuse to eat anything that has been trodden under foot or soiled in any other manner.

The art of milking can be acquired by the most unskilled, city-bred person by the exercise of patience and perseverance: patience on the part of the does and perseverance on the part of the milker. While it naturally requires practice to become an adept, a few lessons from one who knows how will greatly expedite matters and prove less trying to those docile animals. They should be milked at a regular hour night and morning. The directions given by Pegler, in The Book of the Goat, have been found of great assistance to us in our efforts to acquire the "gentle art."

The goat fancier who cares for and milks his goats himself will soon find that an hour or two a day thus spent will prove a very pleasant and satisfying occupation. The care of these "little giant milk producers," aptly described by Hook as "the most intelligent, the most engaging, and most picturesque of domestic cattle," opens an inviting and useful recreation or occupation, not only to men, but to women and even to children, commending itself especially to those whose health requires some light form of outdoor work, either as a vocation or an avocation. A great advantage, from an economic point of view, is that it requires but a small outlay to establish or to maintain a small goat dairy. In fact, there are few undertakings which can be commenced on so small a scale that can be made to pay so well, both in pleasure and profit.

The importance of milch goat keeping cannot fail to appeal to physicians or to parents of young children, for it has been proved beyond refutation that infants deprived of their mother's milk thrive upon goat's milk better than on that of any other animal. The *Lancet* of May 25, 1907, in reporting an analysis of goat's milk made by the *Lancet* laboratory, says that "there are points about goat's milk in connection with infant feeding which deserve more attention than they have hitherto received. It is well known, for example, that the goat is remarkably resistant to tuberculosis; moreover, the milk appears to be more digestible than cow's milk, because its casein forms a flocculent, rather than a hard, cheesy curd. It has been stated, however, that the unpleasant odor of goat's milk is an unfavorable feature. As a matter of fact, goat's milk can be as sweet in this regard as cow's milk so long as the animals are kept under clean and proper conditions. A sample of goat's milk submitted to us was perfectly sweet to the taste and smell, and there was no suggestion at all of the so-called goat flavor. The milk represents the drawing from a mixed herd which is entirely stall fed, the dry foods given in the winter being replaced by a liberal allowance of grass and green stuff in the summer. The animals are carefully groomed and their udders are washed daily.

"On analysis the milk gave the following results: Total solid matter, 14.57 per cent; milk sugar, 5.05 per cent; fat, 5.27 per cent; protein, 3.43 per cent; and mineral matter 0.82 per cent. It will be seen that the milk is of excellent quality, containing a maximum proportion of fat."

## Figures and Tables

**Figure f1:**
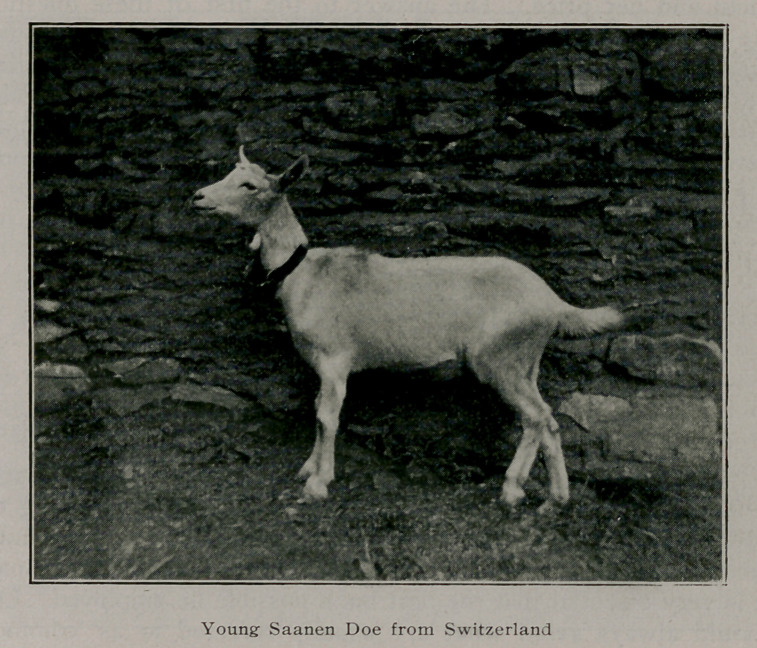


**Figure f2:**